# Effects of dietary *Schizochytrium spp.* alone or combined with *Nannochloropsis gaditana* on milk fatty acid profile and oxidative status in lactating ewes

**DOI:** 10.1093/jas/skag256

**Published:** 2026-08-17

**Authors:** Alexandros Mavrommatis, Fabio Correddu, Panagiota Kyriakaki, Fotis F Satolias, Anna Nudda, Eleni Tsiplakou

**Affiliations:** Laboratory of Nutritional Physiology and Feeding, Department of Animal Science, School of Animal Biosciences, Agricultural University of Athens, Athens 11855, Greece; Dipartimento di Agraria, University of Sassari, Sassari 07100, Italy; Laboratory of Nutritional Physiology and Feeding, Department of Animal Science, School of Animal Biosciences, Agricultural University of Athens, Athens 11855, Greece; Laboratory of Nutritional Physiology and Feeding, Department of Animal Science, School of Animal Biosciences, Agricultural University of Athens, Athens 11855, Greece; Dipartimento di Agraria, University of Sassari, Sassari 07100, Italy; Laboratory of Nutritional Physiology and Feeding, Department of Animal Science, School of Animal Biosciences, Agricultural University of Athens, Athens 11855, Greece

**Keywords:** polyunsaturated fatty acids, biohydrogenation, milk composition, antioxidants, oxidative stress, nutrition

## Abstract

The objective of this study was to evaluate the effects of dietary supplementation with *Schizochytrium spp*., either alone or combined with *Nannochloropsis gaditana*, on milk yield and composition, milk fatty acid profile, and oxidative status in lactating ewes. Thirty-two crossbred dairy ewes in early lactation (50 ± 5 d in milk) were allocated to four groups (*n* = 8 per group) balanced for milk yield, body weight, and age. The control group (CON) received a concentrate without microalgae, whereas the supplemented groups received concentrates providing 30 g/ewe/d of *Schizochytrium spp*. (SC30) or 30 g/ewe/d (MB30) or 40 g/ewe/d (MB40) of a 70:30 blend of *Schizochytrium spp.* and *N. gaditana*. The experiment lasted 90 d, and milk and blood samples were collected on d 18, 36, 54, 72, and 90. Dietary treatment did not affect milk yield, fat-corrected milk (FCM), energy-corrected milk (ECM), milk fat yield, or milk protein yield (*P *> 0.10), although milk fat percentage tended to be lower in MB30 than in CON (*P *= 0.083). Milk C18:0 and cis-9 C18:1 concentrations were lower in all supplemented groups than in CON (*P *= 0.006 and *P *< 0.001, respectively). Trans-11 C18:1 and cis-9, trans-11 CLA were greater in SC30 and MB40 than in CON and MB30 (*P *= 0.012 and *P *= 0.015, respectively). Milk C20:5 n-3 increased in all supplemented groups and was greatest in MB40 (*P *< 0.001), whereas C22:6 n-3 was greater in all supplemented groups than in CON, with the greatest concentrations observed in SC30 and MB40 and an intermediate concentration in MB30 (*P *< 0.001). Similarly, total polyunsaturated fatty acids (PUFA) and n-3 PUFA were greater in SC30 and MB40 than in CON, with MB30 showing intermediate values (*P *< 0.001), whereas the n-3/n-6 ratio increased in all supplemented groups compared with CON (*P *< 0.001). In plasma, catalase activity tended to be greatest in MB40 (*P *= 0.066), glutathione transferase activity was greatest in MB40 and higher than in SC30, with CON and MB30 showing intermediate values (*P *= 0.036), and protein carbonyl concentrations were lower in MB30 than in CON and SC30 (*P *= 0.015). In milk, ABTS radical-scavenging capacity and glutathione peroxidase activity were lower in all supplemented groups than in CON (*P *= 0.003 and *P *= 0.043, respectively). Overall, microalgae supplementation, particularly treatments supplying greater amounts of *Schizochytrium spp*. (SC30 and MB40), enriched ewe milk with long-chain PUFA without impairing milk yield or major production traits.

## Teaser

Feeding dairy ewes with microalgae enriched milk with long-chain polyunsaturated fatty acids without reducing milk yield. However, the response depended on the microalgae source and inclusion level, and changes in saturated fatty acids and oxidative-status biomarkers should also be considered.

## Introduction

Omega-3-enriched functional foods represent one of the fastest-growing sectors globally, driven by increasing consumer awareness of their cardiovascular and cognitive health benefits (Vignesh et al. 2024). Within the dairy sector, milk and dairy products enriched with n-3 fatty acids have received increasing research and commercial interest as functional foods, although their contribution to total dairy production remains limited (Gebreyowhans et al. 2019). Consequently, the development of innovative strategies to enhance the polyunsaturated fatty acid (PUFA) content of foods is of increasing interest. Microalgae represent a renewable and sustainable source of bioactive compounds, including long-chain n-3 PUFA and antioxidants (Mavrommatis et al. 2023). In particular, microalgae-derived lipids such as docosahexaenoic acid (DHA), eicosapentaenoic acid (EPA), and docosapentaenoic acid (n-6 DPA) are of high nutritional value. Dietary supplementation of ruminants with *Schizochytrium spp*., a DHA-rich microalga, has been shown to effectively enrich milk from cows (Franklin et al. 1999), goats (Mavrommatis and Tsiplakou 2020; Pajor et al. 2021), and ewes (Bichi et al. 2013; Toral et al. 2010; Zisis et al. 2022).

Although PUFA-rich feeds effectively enrich dairy products with bioactive lipids, their practical on-farm application raises critical considerations. High PUFA inclusion can alter rumen microbial populations and consequently affect biohydrogenation (Maia et al. 2010). This partial inhibition promotes the accumulation of biohydrogenation intermediates such as trans-10, cis-12 C18:2 and trans-10 C18:1 (Baumgard et al. 2000). Such intermediates can inhibit milk-fat synthesis (Bauman and Griinari 2001; Shingfield et al. 2010), leading to milk-fat depression (MFD), a major challenge for the dairy industry. The severity of MFD depends on species (Carreño et al. 2016), dietary fiber-to-starch ratio (Shingfield et al. 2010), and PUFA level and profile (Mavrommatis and Tsiplakou 2020). A second concern is the high oxidative susceptibility of PUFA, attributed to their multiple double bonds (Decker et al. 2012). This promotes the formation of reactive oxygen species (ROS) and oxidative metabolites, indicators of oxidative stress (Tao 2015). Both the source and inclusion level of PUFA influence tissue oxidative status, as shown in rats (Yuan and Kitts 2003), goats (Mavrommatis et al. 2018), and sheep (Zisis et al. 2022). In our previous studies, in goats, supplementation with *Schizochytrium spp.* enriched milk with n-6 DPA and DHA; however, daily doses above 40 g per ewe caused oxidative stress, reduced milk oxidative stability, and induced MFD (Mavrommatis et al. 2018; Mavrommatis and Tsiplakou 2020). Similarly, more than 30 g *Schizochytrium spp.* in ewe diets induced MFD and impaired oxidative stability in both milk and animals (Zisis et al. 2022). Although reducing dietary PUFA could mitigate MFD and oxidative imbalances, it would also limit milk fortification due to the moderate transfer efficiency of these fatty acids from feed to milk (Farina et al. 2015; Toral et al. 2017; Pajor et al. 2019; Mavrommatis and Tsiplakou 2020). This limitation is particularly relevant from a regulatory perspective, as under EU Regulation 116/2010, a food can be labeled as a source of omega-3 only if it provides ≥40 mg EPA + DHA per 100 g and per 100 kcal (European Commission 2010).

One possible strategy to address these limitations is to combine a PUFA-rich microalga with another microalga containing antioxidant and bioactive compounds, aiming to enrich milk with long-chain n-3 PUFA while limiting adverse effects on ruminal lipid metabolism, milk fat synthesis, and oxidative status. Among candidate species, *Schizochytrium spp.* are rich in DHA and n-6 DPA, while *Nannochloropsis spp.* provide abundant EPA (El-Sayed et al. 2024). Beyond lipids, *Nannochloropsis* species contain diverse antioxidant compounds, including flavonoids, β-carotene, phenolic acids, and triterpenoids, conferring strong antioxidant capacity (Emam et al. 2024). Recent evidence also indicates that *Nannochloropsis spp.* act as functional feed ingredients by modulating microbiota and fermentation; *in vitro* digests of *N. gaditana* increased short-chain fatty acid (SCFA)-producing bacteria and SCFA (Paterson et al. 2023) and have been proposed as prebiotics enhancing rumen fermentation (Saadaoui et al. 2021; El-Hawy et al. 2022). We hypothesize that combining *Schizochytrium spp*. with *N. gaditana* enriches ewe milk with the dominant fatty acids supplied by each microalga, namely DHA from *Schizochytrium spp*. and EPA from *N. gaditana*, while attenuating the risk of MFD and oxidative imbalance commonly associated with marine PUFA supplementation. We further hypothesize that these responses depend on the inclusion level of the microalgae blend. Accordingly, this study aimed to evaluate the effects of dietary supplementation with *Schizochytrium spp*., alone or combined with *N. gaditana*, on milk yield and composition, milk fatty acid profile and oxidative stability, and ewe oxidative status.

## Material and methods

Animal handling procedures were performed following protocols approved by the Agricultural University of Athens Ethical Committee (approval no. 31/27-05-2021). The study was conducted with respect to the guidelines of the European Union Directive 2010/63/EU on the protection of animals used for scientific purposes.

### Animals and diets

Thirty-two crossbred dairy ewes [Lacaune × Local (Greek) breed] of comparable age (3-4 years old) and body weight (74 ± 4 kg), at early lactation (50 ± 5 d in milk) were used. The ewes were separated into four homogeneous groups (*n* = 8 per group) according to their milk yield (Fat Corrected Milk, FCM 6%; 2.73 ± 0.433 kg), body weight and age. Each ewe was offered 2 kg of alfalfa hay and 2 kg of concentrate daily (F:C = 50:50), divided into two equal meals (08:00 and 20:00 h), and was fed individually throughout the experimental period. The concentrates supplied to the control (CON) group had no microalgae while those of the treated groups were supplemented daily with 30 g *Schizochytrium spp.* (SC30) or 30 g (MB30) and 40 g (MB40) with microalgae blend of *Schizochytrium spp./N. gaditana* at a 70:30 ratio (MB30: 21 g *Schizochytrium spp.* and 9 g *N. gaditana*; MB40: 28 g *Schizochytrium spp.* and 12 g *N. gaditana*). Microalgae supplementation levels were selected based on previous dose-response studies in sheep (Zisis et al. 2022) and goats (Mavrommatis et al. 2018), in which similar or higher inclusion levels of *Schizochytrium spp*. were evaluated in relation to milk fatty acid enrichment, MFD, and oxidative status. The 70:30 ratio between *Schizochytrium spp*. and *N. gaditana* was selected based on preliminary unpublished data indicating the minimum inclusion of *N. gaditana* required to provide a measurable EPA contribution to the milk fatty acid profile while maintaining *Schizochytrium spp*. as the main DHA-rich component of the blend. The same supplementation scheme was also used in a companion study evaluating antioxidant- and immune-related gene expression in ewes’ monocytes and neutrophils (Mavrommatis et al. 2026). Microalgae were incorporated into the concentrate by partially replacing soybean oil and palm oil in the control (CON) formulation in order to achieve isoenergetic diets with comparable ether extract (EE) levels, without markedly altering the overall ingredient composition (Table 1). NRC (2007) was used to predict the nutrient requirements of dairy ewes. Refusals were recorded daily and pooled weekly for chemical analysis to determine the actual nutrient intake after forage sorting. The *Schizochytrium spp.* used was a commercial product (DHA GOLD S17-B, DSM Nutritional Products, Marousi, Greece), while *N. gaditana* was cultivated for the needs of the trial (Mikrophykos, Athens, Greece). The concentrate diets were prepared every 20 d and kept under the appropriate conditions in order to prevent any oxidation of the microalgae. The experimental period lasted 100 d with the first 10 d considered an adaptation period for the animals in the pens and feeders.

**Table 1:** Concentrate ingredient composition.

	CON	SC30	MB30	MB40
**Item (in g/kg as fed)**				
**Maize grain**	472.0	469.5	469.5	464.5
**Sunflower meal**	80	80	80	80
**Wheat middling**	70	70	70	70
**Barley**	100	100	100	100
**Soyabean meal**	185	185	185	185
**Calcium carbonate**	14	14	14	14
**Sodium bicarbonate^1^**	3	3	3	3
**Premix of minerals and vitamins^2^**	25	25	25	25
**Mycotoxin binder^3^**	1	1	1	1
**Dried brewer’s yeast**	25	25	25	25
**Soyabean oil**	5	2.5	2.5	2.5
**Palm oil^4^**	20	10	10	10
***Schizochytrium spp.*^5^**	—	15	10.5	14
***Nannochloropsis gaditana*^6^**	—	—	4.5	6

CON: concentrate mix without microalgae; SC30: 30 g microalgae *Schizochytrium spp*./ewe/d; MB30: 30 microalgae blend g/ewe/d (21 g *Schizochytrium spp*. and 9 g *Nannochloropsis gaditana*); MB40: 40 microalgae blend g/ewe/d (28 g *Schizochytrium spp*. and 12 g *Nannochloropsis gaditana*).

1 Bicar Z 0/50 Sodium Bicarbonate.

2 Premix of minerals and vitamins provides, per kg of product: vitamin A (E672), 480,000 IU; vitamin D_3_ (E671), 80,000 IU; vitamin E (E307), 1,200 IU; iodine (as calcium iodate, anhydrous), 40 mg; cobalt (as cobalt sulfate), 12 mg; niacin, 800 mg; iron (as iron carbonate), 2,400 mg; manganese (as manganese oxide), 1,600 mg; selenium (as sodium selenite), 8 mg; zinc (as chelated zinc), 300 mg. An aromatic additive (LUCTAROM) is also included.

3 TOXFIN® Dry based on specially activated aluminosilicate components.

4 Bergafat T-300 is derived from fractionated palm oil and contains its natural glycerol.

5 DHAgold™ (DSM; DHAgold S17-B).

6 Cultivated for the needs of the trial by Mikrophykos, Athens, Greece.

Samples of microalgae and alfalfa hay were collected before the experimental period for nutritional assessment, whereas concentrate samples were collected every 20 d, coinciding with the preparation of new batches. Refusals were collected as described above and analyzed at the end of the trial. Samples were analyzed for crude protein (CP; Official Method 7.016) according to the Association of Official Analytical Chemists (AOAC 1984), using a Kjeldahl Distillation System (FOSS Kjeltec 8400, Hillerød, Denmark). EE was determined according to AOAC Official Method 920.39 (AOAC 2005a), and dry matter (DM) was measured by drying samples at 103 °C overnight according to AOAC Method 7.007 (AOAC 1984). Neutral detergent fiber (aNDF) in alfalfa hay and concentrates was analyzed according to AOAC Official Method 2002.04, as described by Mertens (2002), whereas acid detergent fiber (ADF) was determined according to AOAC Official Method 973.18 (AOAC 2005b). The aNDF was determined using heat-stable amylase, without sodium sulfite, and expressed inclusive of residual ash. Feeds were also analyzed for fatty acid composition according to the method of O’Fallon et al. (2007), using an Agilent 6890 N gas chromatograph equipped with an HP-88 capillary column (60 m × 0.25 mm i.d., 0.20 µm film thickness; Agilent Technologies, Inc., Santa Clara, CA, USA) and a flame ionization detector, as described by Mavrommatis and Tsiplakou (2020).

### Milk collection and chemical composition

The ewes were milked twice a day at 07:30 and 19:30 by a portable milking machine. Individual milk samples were collected into plastic tubes at 18, 36, 54, 72, and 90 d of the main experimental period for the determination of chemical composition, fatty acid profile, total antioxidant capacity, antioxidant enzyme activities, and oxidative biomarkers after pooling 5% of the yield from the evening and morning milkings. Fresh milk samples were analyzed for fat, protein, lactose, total solids, and solids-not-fat by IR spectrometry (MilkoScan 120; Foss Electric, Hillerød, Denmark) after proper calibration. FCM yield (6%) was calculated according to Bocquier et al. (1993) as: FCM = milk yield × [0.28 + 0.12 × fat (%)]. ECM yield was calculated as: ECM = milk yield × [0.071 × fat (%) + 0.043 × protein (%) + 0.2224], as described by Bocquier et al. (1993). The milk samples were stored at −80 °C, before the analyses.

### Blood plasma preparation

The blood samples were collected from the jugular vein and put into 17 Units/mL heparin-containing tubes (BD Vacutainer, Plymouth, UK) at 18, 36, 54, 72, and 90 d of the main experimental period for the determination of the total antioxidant capacity, antioxidant enzyme activities, and oxidative biomarkers. After the collection, the blood samples were subsequently centrifuged at 2700 × g for 10 min to separate plasma from the cells. Thereafter, plasma samples were stored at −80 °C, prior to analyses.

### Enzyme assays, oxidative stress biomarkers, and total antioxidant capacity

The measurement of the enzyme activities (superoxide dismutase; SOD, catalase; CAT, glutathione reductase; GR, glutathione peroxidase; GSH-Px, glutathione transferase; GST), the oxidative stress biomarkers (malondialdehyde; MDA, protein carbonyls; PC), and the total antioxidant capacity (Ferric Reducing Antioxidant Power; FRAP), 2,2′-azino-bis (3 ethylbenzthiazoline-6-sulfonic acid; ABTS) were based on the principles of spectrophotometry and conducted as described by Tsiplakou et al. (2017). For GST activity, plasma was mixed with potassium phosphate buffer (pH 6.5) containing reduced glutathione and 1-chloro-2,4-dinitrobenzene, and the formation of the glutathione-conjugated product was monitored at 340 nm for 2 min. CAT activity in plasma and milk was assessed using H_2_O_2_ as substrate, by continuously recording the decrease in absorbance associated with H_2_O_2_ decomposition at 520 nm. GSH-Px activity in plasma and milk was measured in potassium phosphate buffer (pH 7.0) containing EDTA, reduced glutathione, H_2_O_2_, NADPH, and glutathione reductase; enzyme activity was calculated from the rate of NADPH oxidation recorded at 340 nm for 5 min. GR activity in plasma and milk was determined in potassium phosphate buffer (pH 7.6) containing oxidized glutathione, NADPH, bovine serum albumin, and double-distilled water by monitoring the decrease in absorbance at 340 nm for 3 min. SOD activity in plasma and milk was assayed in a reaction cocktail containing potassium phosphate buffer, EDTA, cytochrome c, xanthine, and xanthine oxidase; activity was determined from the inhibition of cytochrome c reduction, with absorbance recorded at 550 nm for 5 min. Total antioxidant capacity was evaluated using FRAP and ABTS assays. For FRAP, samples were mixed with acetate buffer, 2,4,6-Tri(2-pyridyl)-s-triazine (TPTZ), and ferric chloride reagent, incubated at 37 °C for 4 min, and absorbance was measured at 593 nm. For ABTS, the ABTS radical cation was prepared with potassium persulfate and diluted with phosphate-buffered saline to an absorbance of 0.70 at 734 nm. Diluted samples were then mixed with the ABTS working solution, and absorbance was measured at 734 nm; results were expressed as percentage inhibition. For milk FRAP and ABTS analyses, 1 mL of fresh milk was extracted with 10 mL of HCl/ethanol solution, shaken for 1 h at 30 °C, centrifuged at 7,800 × g for 15 min at 5 °C, and the supernatant was used for analysis. Lipid peroxidation was assessed by measuring MDA using the thiobarbituric acid-reactive substances assay. Plasma samples were mixed with 1% orthophosphoric acid and 0.6% thiobarbituric acid, whereas milk samples were mixed with the same reagents at a 1:7:2 ratio of milk, orthophosphoric acid, and thiobarbituric acid. Samples were heated at 100 °C for 60 min, filtered through 0.45-μm cellulose acetate filters, and absorbance was recorded at 532 nm. Protein carbonyls were determined after mixing plasma or milk with trichloroacetic acid, incubating on ice for 15 min, and centrifuging at 15,000 × g for 5 min at 4 °C. The precipitate was derivatized with 2,4-dinitrophenylhydrazine for 1 h in the dark, washed with trichloroacetic acid and ethanol/ethyl acetate, dissolved in urea, incubated at 37 °C for 15 min, centrifuged again, and absorbance was read at 375 nm. PC concentration was calculated using the molar extinction coefficient of 2,4-dinitrophenylhydrazine (DNPH).

### Milk fatty acid determination

The fatty acid profile of milk samples was also examined according to the method of Nourooz-Zadeh and Appelqvist (1998) as described by Tsiplakou et al. (2006). Milk samples were analyzed for fatty acid methyl ester (FAME) following Carta et al. (2022). A 7890A GC System (Agilent Technologies, Santa Clara, CA, USA) was used to determine the FA profile with a capillary column CP-SIL 88 for FAME (100 m × 0.25 mm i.d., 0.29 μm film thickness, Agilent Technologies). The instrument was equipped with a 7693 Autosampler (Agilent Technologies) and a flame ionization detector. Helium was used as the carrier gas at a flow rate of 1 mL/min and a pressure of 28 psi. The initial oven temperature was 45 °C, maintained for 4 min; then, the temperature was increased by 13 °C/min until 175 °C and it was maintained for 27 min. After that, the temperature increased by 4 °C/min until 215 °C. The temperatures of the injector and detector were 250 °C. The split ratio was 1:80. The program used to determine the area of the FAME was the OpenLAB CDS GC ChemStation Upgrade software data system (Revision C.01.04, Agilent Technologies Inc., Santa Clara, CA, USA). The peaks were identified by comparing their retention time with those of the methyl standard, as detailed in Nudda et al. (2005).

### Statistical analysis

Data were analyzed using IBM SPSS Statistics (version 26.0) and are presented as estimated marginal means (least-squares means; LS-means) with their standard errors (SEM). Dietary effects were evaluated using a linear mixed model for repeated measures. Dietary treatment (D: CON, SC30, MB30, and MB40), sampling time (S: days 18, 36, 54, 72, and 90 of the experimental period), and their interaction (D × S) were included as fixed effects. Animals were included as a random effect, and sampling time was specified as a repeated measure within animals. The covariance structure for repeated observations was selected by comparing candidate structures using information criteria and model convergence. For milk yield and chemical composition variables, the corresponding measurement obtained on d 0 was included as a covariate to adjust for initial differences among animals. D 0 covariates were not included in the analyses of milk fatty acid profile or oxidative status variables because these measurements were not available before supplementation. When main effects were detected at *P *< 0.10, pairwise comparisons among dietary-treatment and sampling-time marginal means were performed separately using Fisher’s least significant difference test. Effects were considered significant at *P *< 0.05, whereas 0.05 ≤ *P *< 0.10 was discussed as a tendency.

## Results

### Feed intake

The chemical analysis revealed distinct compositional profiles between the two microalgae species (Table 2). *Schizochytrium spp.* exhibited higher EE and was particularly rich in DHA (C22:6 n-3), whereas *N. gaditana* contained greater crude protein and was characterized by elevated EPA (C20:5 n-3) concentration. Table 3 presents the daily nutrient intake of sheep fed experimental diets throughout the study period. The dietary treatments did not affect (*P *> 0.05) the intake of DM, ash, CP, ADF, or aNDF. In contrast, EE intake was significantly influenced by diet (*P *< 0.001). Regarding fatty acid intake, most individual fatty acids were affected by dietary treatment due to microalgae supplementation (Table 3). The intakes of myristic (C14:0), palmitic (C16:0), oleic (cis-9 C18:1), linoleic (C18:2 n-6), eicosapentaenoic (EPA; C20:5 n-3), docosapentaenoic (n-6 DPA; C22:5 n-6), and docosahexaenoic (DHA; C22:6 n-3) acids differed significantly among groups (*P *≤ 0.001). The control diet resulted in greater intakes of C16:0, cis-9 C18:1, and C18:2 n-6 compared with the microalgae-supplemented treatments (*P *< 0.001). Intake of C14:0 was higher in SC30 and MB40 than in MB30 and CON. EPA intake increased only in the blend treatments, with the greatest value observed in MB40. In contrast, n-6 DPA and DHA intakes were increased in all microalgae-supplemented groups compared with CON, with the highest values observed in SC30 and the next highest values in MB40 (*P *< 0.001).

**Table 2:** Chemical composition and fatty acid profile of *Schizochytrium spp.* and *Nannochloropsis gaditana.*

	*Schizochytrium spp.*	*Nannochloropsis gaditana*
**Item as g/kg as fresh**		
**Dry matter**	980	915
**Crude protein**	167	410
**Ether extract**	556	100
**Ash**	88	100
**% of total fatty acids**		
**C14:0**	5.9	4.5
**C16:0**	13.1	18.3
**C18:0**	0.30	0.2
**cis-9 C18:1**	—	4.7
**C18:3 n-3**	0.1	—
**C20:5 n-3**	—	34.5
**C22:5 n-6**	6.8	—
**C22:6 n-3**	21.8	—

**Table 3:** Nutrient daily intake of the experimental diet throughout the experimental period.

Item (in g/d except as noted)	Dietary treatment^1^					
	CON	SC30	MB30	MB40	SEM^2^	*P*-value
**DM**	3404.2	3458.6	3432.1	3473.0	10.478	0.102
**Ash**	248.9	250.5	250.7	253.4	0.689	0.142
**CP**	599.1	603.5	600.4	607.0	1.246	0.101
**Ether extract**	137.6^a^	135.2^b^	132.5^c^	135.4^b^	0.349	<0.001
**aNDF**	1142.0	1126.0	1134.0	1138.0	6.003	0.191
**ADF**	693.0	705.4	702.5	718.6	4.187	0.192
**Fatty acids intake in g/d**						
**C14:0**	2.67^c^	3.55^a^	3.29^b^	3.54^a^	0.156	0.001
**C16:0**	49.29^a^	46.82^b^	46.33^c^	46.89^b^	2.394	<0.001
**C18:0**	4.80	4.30	4.28	4.29	0.220	0.108
**cis-9 C18:1**	18.40^a^	13.90^b^	13.94^b^	13.95^b^	0.777	<0.001
**C18:2 n-6**	46.24^a^	43.89^b^	43.89^b^	43.89^b^	2.224	<0.001
**C18:3 n-3**	13.37	13.54	13.46	13.58	0.669	0.101
**C20:5 n-3**	0.03^c^	0.03^c^	0.34^b^	0.44^a^	0.011	<0.001
**C22:5 n-6**	0.02^d^	1.15^a^	0.81^c^	1.08^b^	0.038	<0.001
**C22:6 n-3**	0.00^d^	3.64^a^	2.55^c^	3.39^b^	0.120	<0.001

1 CON: concentrate mix without microalgae; SC30: 30 g microalgae *Schizochytrium spp*./ewe/d; MB30: 30 microalgae blend g/ewe/d (21 g *Schizochytrium spp*. and 9 g *Nannochloropsis gaditana*); MB40: 40 microalgae blend g/ewe/d (28 g *Schizochytrium spp*. and 12 g *Nannochloropsis gaditana*).

2 SEM = Standard error of the mean.

Different lowercase superscript letters (a, b, c, d) indicate significant differences (*P *< 0.05) among dietary treatments.

### Milk yield and chemical composition

Dietary supplementation with microalgae did not significantly affect milk yield, FCM, or ECM yield (*P *> 0.10; Table 4). Likewise, the daily outputs of milk protein and fat were not influenced by treatment (*P *= 0.741 and *P *= 0.157, respectively). There was a tendency for a decrease in milk fat percentage in ewes receiving the MB30 diet compared with CON (*P *= 0.083), whereas SC30 and MB40 showed intermediate values that did not differ from either group. The milk protein, lactose, and total solids-not-fat contents of milk were not significantly influenced by dietary treatment.

**Table 4:** Milk yield and composition of lactating ewes fed diets supplemented with microalgae.

	Dietary group^1^ (D)					Sampling time (T)						Effect		
	CON	SC30	MB30	MB40	SEM^2^	18	36	54	72	90	SEM^2^	D	T	D × T
**Yield, g/d**														
**Milk**	2284	2202	2138	2275	135.78	2309^a^	2328^a^	2345^a^	2096^b^	2045^b^	88.75	0.859	<0.001	0.991
**FCM**	2470	2198	2107	2270	124.72	2424^a^	2351^a,b^	2382^a^	2203^b^	1946^c^	74.23	0.285	<0.001	0.979
**ECM**	2198	2017	1920	2056	110.39	2158^a^	2117^a,b^	2160^a^	1993^b^	1810^c^	71.86	0.394	<0.001	0.991
**Fat**	151.7	131.7	125.9	136.7	7.46	148.1^a^	141.8^a,b^	143.5^a,b^	134.7^b^	114.3^c^	5.12	0.157	<0.001	0.958
**Protein**	139.9	137.1	129.8	136.1	6.59	138.1^a,b^	138.4^a,b^	143.6^a^	132.7^b,c^	126.1^c^	4.39	0.741	0.007	0.997
**Milk composition, g/100 g**														
**Fat**	6.77^e^	6.07^e,f^	5.89^f^	6.11^e,f^	0.230	6.43^a^	6.17^b^	6.15^b^	6.55^a^	5.74^c^	0.141	0.083	0.002	0.174
**Protein**	6.27	6.32	6.14	5.96	0.150	6.02^c^	6.00^c^	6.17^b^	6.43^a^	6.25^b^	0.092	0.399	0.002	0.169
**Lactose**	4.54	4.62	4.55	4.59	0.053	4.58^b,c^	4.66^a^	4.60^b^	4.49^c^	4.55^b,c^	0.026	0.700	<0.001	0.883
**Total solids**	18.20	17.58	17.07	17.25	0.330	17.57^b^	17.41^b^	17.49^b^	18.03^a^	17.13^b^	0.187	0.149	0.005	0.133
**Total solid no fat**	11.63	11.71	11.47	11.40	0.154	11.38^c^	11.49^b^	11.58^a,b^	11.72^a^	11.60^a,b^	0.093	0.417	0.010	0.243

1 CON: concentrate mix without microalgae; SC30: 30 g microalgae *Schizochytrium spp*./ewe/d; MB30: 30 microalgae blend g/ewe/d (21 g *Schizochytrium spp*. and 9 g *Nannochloropsis gaditana*); MB40: 40 microalgae blend g/ewe/d (28 g *Schizochytrium spp*. and 12 g *Nannochloropsis gaditana*).

2 SEM = Standard error of the mean.

Values are least squares means derived from a repeated-measures model. Measurements were obtained at five consecutive sampling times (d 18, 36, 54, 72, and 90 of the experimental period). Within each row, different lowercase superscript letters within the dietary treatment columns indicate differences among dietary treatments, whereas different lowercase superscript letters within the sampling time columns indicate differences among sampling days (*P* < 0.05). Dietary treatment means and sampling time means were compared separately. Lowercase superscript letters e and f indicate tendencies (0.05 ≤ *P* < 0.10) within the corresponding main effect. FCM: Fat corrected milk in 6% according to the equation Y6% = (0.28 + 0.12F) M, where F = fat% and M = milk yield in g; ECM: Energy corrected milk = milk yield × (0.071 × fat (%) + 0.043 × protein (%) + 0.2224).

### Milk fatty acid profile

Microalgae supplementation significantly modified several individual SFA (Table 5). SCFA such as C6:0 (*P *= 0.002) and C8:0 (*P *< 0.001) were higher in SC30 and MB40 than in CON and MB30. Likewise, C10:0 (*P *= 0.020) increased in MB40 and SC30 compared with CON, while C14:0 tended to be lower in CON than in the microalgae-supplemented groups (*P *= 0.097). Conversely, C18:0 was markedly reduced in all groups supplemented with microalgae (*P *= 0.006). C16:0 differed among the supplemented groups (*P *= 0.009), with the highest value in MB30 compared with SC30 and MB40. None of the supplemented groups differed from CON for C16:0. Among long-chain FA, C20:0 (*P *= 0.031) was decreased and C22:0 increased by SC30 and MB30 supplementation compared to CON, whereas C23:0 (*P *= 0.001) was increased only by MB30 compared to CON. Among the branched-chain FA the C17:0 *iso* was increased (*P *= 0.005) in MB40 relative to other groups, whereas iso-C13:0 tended to increase in MB30 compared with CON (*P *= 0.093).

**Table 5:** Saturated fatty acid composition (g/100 g FA) of milk from lactating ewes fed diets supplemented with microalgae.

	Dietary group^1^ (D)					Sampling time (T)						Effect		
	CON	SC30	MB30	MB40	SEM^2^	18	36	54	72	90	SEM^2^	D	T	D × T
**C4:0**	4.31	4.38	4.23	4.26	0.173	4.44^a^	4.39^a,b^	4.27^b,c^	4.23^c^	4.14^d^	0.088	0.920	0.005	<0.001
**C6:0**	2.67^b^	3.07^a^	2.85^b^	3.15^a^	0.084	3.07^a^	3.03^a^	2.99^a^	2.89^b^	2.69^c^	0.046	0.002	<0.001	0.671
**C7:0**	0.035	0.036	0.037	0.038	0.002	0.040^b^	0.046^a^	0.027^c^	0.030^c^	0.039^b^	0.002	0.431	<0.001	0.061
**C8:0**	2.26^b^	2.78^a^	2.47^b^	2.95^a^	0.091	2.77^a^	2.72^a^	2.69^a^	2.58^b^	2.33^c^	0.055	<0.001	<0.001	0.830
**C9:0**	0.072	0.063	0.062	0.066	0.005	0.069^b^	0.062^b,c^	0.061^b,c^	0.081^a^	0.056^c^	0.004	0.400	<0.001	0.044
**C10:0**	8.00^c^	9.15^a,b^	8.41^b,c^	9.58^a^	0.362	9.43^a^	9.10^a,b^	8.94^b,c^	8.74^c^	7.70^d^	0.212	0.020	<0.001	0.400
**C11:0**	0.390	0.426	0.450	0.444	0.029	0.468^a^	0.443^a,b^	0.425^b^	0.435^b^	0.367^c^	0.017	0.474	<0.001	0.002
**C12:0**	5.22	5.52	5.14	5.78	0.273	5.77^a^	5.53^b^	5.48^b^	5.46^b^	4.84^c^	0.155	0.345	<0.001	0.225
**C13:0**	0.200	0.163	0.166	0.159	0.015	0.215^a^	0.203^a^	0.218^a^	0.138^b^	0.087^c^	0.009	0.205	<0.001	0.032
**C14:0**	12.16^f^	12.97^e^	13.22^e^	12.91^e^	0.300	12.96^a^	12.61^b^	12.93^a^	13.14^a^	12.43^b^	0.162	0.097	<0.001	0.248
**C15:0**	1.04	0.90	0.93	0.90	0.044	1.00^a^	0.98^a,b^	0.94^b^	0.87^c^	0.92^b^	0.026	0.226	<0.001	0.808
**C16:0**	28.03^a,b^	27.38^b^	29.59^a^	26.28^b^	0.633	27.51^b^	27.60^b^	27.54^b^	28.93^a^	27.52^b^	0.345	0.009	<0.001	0.376
**C17:0**	0.546	0.495	0.496	0.510	0.021	0.691^a^	0.521^b^	0.439^d^	0.435^d^	0.472^c^	0.015	0.273	<0.001	<0.001
**C18:0**	7.44^a^	5.19^b^	5.75^b^	5.30^b^	0.461	5.74^a^	6.24^a^	5.95^a^	5.30^b^	6.36^a^	0.310	0.006	0.003	0.001
**C20:0**	0.164^a^	0.142^b^	0.141^b^	0.156^a,b^	0.006	0.226^a^	0.235^a^	0.134^b^	0.066^d^	0.092^c^	0.006	0.031	<0.001	<0.001
**C22:0**	0.056^b^	0.080^a^	0.080^a^	0.052^b^	0.004	0.140^a^	0.109^b^	0.028^c^	0.024^c^	0.034^c^	0.005	<0.001	<0.001	<0.001
**C23:0**	0.021^bc^	0.027^b^	0.041^a^	0.017^c^	0.004	0.002^d^	0.008^d^	0.062^a^	0.023^c^	0.038^b^	0.004	0.001	<0.001	0.002
**C24:0**	0.036^b^	0.042^a^	0.041^a^	0.033^b^	0.002	0.056^a^	0.051^a^	0.029^b,c^	0.024^c^	0.030^b^	0.002	0.001	<0.001	<0.001
**C25:0**	0.047^a^	0.042^a,b^	0.032^c^	0.040^b^	0.003	0.035^b^	0.035^b^	0.038^b^	0.036^b^	0.058^a^	0.002	0.002	0.001	<0.001
**C26:0**	0.023^a,b^	0.026^a^	0.020^b,c^	0.017^c^	0.002	0.026^a^	0.016^b^	0.024^a^	0.018^b^	0.023^a^	0.002	0.014	0.002	0.140
**C13:0 *iso***	0.045^f^	0.046^f^	0.053^e^	0.048^e,f^	0.003	0.027^d^	0.046^c^	0.068^a^	0.054^b^	0.044^c^	0.002	0.093	<0.001	<0.001
**C13:0 *anteiso***	0.069	0.068	0.073	0.069	0.006	0.068^b^	0.062^c^	0.074^a^	0.079^a^	0.067^b^	0.003	0.945	<0.001	0.381
**C14:0 *iso***	0.083	0.073	0.079	0.079	0.007	0.089^a^	0.078^b^	0.077^bc^	0.072^c^	0.077^b,c^	0.004	0.782	0.001	0.946
**C15:0 *iso***	0.173	0.164	0.171	0.173	0.007	0.184^a^	0.176^b^	0.165^c^	0.149^d^	0.176^b^	0.005	0.796	<0.001	0.190
**C15:0 *anteiso***	0.371	0.315	0.324	0.331	0.018	0.376^a^	0.354^b^	0.309^c^	0.297^c^	0.340^b^	0.010	0.163	<0.001	0.051
**C16:0 *iso***	0.200	0.203	0.210	0.219	0.012	0.236^a^	0.201^b^	0.206^b^	0.187^c^	0.210^b^	0.007	0.701	<0.001	0.260
**C17:0 *iso***	0.203^b^	0.216^b^	0.206^b^	0.264^a^	0.012	0.308^a^	0.257^b^	0.163^d^	0.166^d^	0.217^c^	0.009	0.005	<0.001	0.003
**C17:0 *anteiso***	0.601	0.545	0.688	0.604	0.048	0.257^d^	0.265^d^	0.648^c^	0.998^a^	0.881^b^	0.030	0.215	<0.001	<0.001
**C18:0 *iso***	0.047	0.046	0.044	0.045	0.002	0.043^b^	0.054^a^	0.048^b^	0.037^c^	0.045^b^	0.002	0.801	0.002	0.077

1 CON: concentrate mix without microalgae; SC30: 30 g microalgae *Schizochytrium spp*./ewe/d; MB30: 30 microalgae blend g/ewe/d (21 g *Schizochytrium spp*. and 9 g *Nannochloropsis gaditana*); MB40: 40 microalgae blend g/ewe/d (28 g *Schizochytrium spp*. and 12 g *Nannochloropsis gaditana*).

2 SEM = Standard error of the mean.

Values are least squares means derived from a repeated-measures model. Measurements were obtained at five consecutive sampling times (d 18, 36, 54, 72, and 90 of the experimental period). Within each row, different lowercase superscript letters within the dietary treatment columns indicate differences among dietary treatments, whereas different lowercase superscript letters within the sampling time columns indicate differences among sampling days (*P* < 0.05). Dietary treatment means and sampling time means were compared separately. Lowercase superscript letters e and f indicate tendencies (0.05 ≤ *P* < 0.10) within the corresponding main effect.

Diet significantly affected several MUFA (Table 6). The inclusion of microalgae induced a reduction in some cis MUFA, accompanied by a concomitant accumulation of several trans-isomers. Specifically, almost all trans C18:1 isomers detected were significantly increased by SC30 and MB40 supplementation. Among these, trans-10 C18:1 was increased in SC30 and MB40 compared with CON and MB30 (*P *= 0.004) and showed a significant diet × sampling time interaction (*P *= 0.001). In particular, the main trans FA, trans-11 C18:1, reached the highest concentrations with SC30 and MB40 (*P *= 0.012). In contrast, cis-9 C18:1 (oleic acid) decreased sharply (*P *< 0.001) in all supplemented groups. Minor cis isomers such as cis-11 C18:1 and cis-16-14 C18:1 also varied significantly.

**Table 6:** Monounsaturated fatty acid profile (g/100 g FA) of the milk from lactating ewes fed microalgae-supplemented diets.

	Dietary group^1^ (D)					Sampling time (T)						Effect		
	CON	SC30	MB30	MB40	SEM^2^	18	36	54	72	90	SEM^2^	D	T	D × T
***cis-9* C10:1**	0.155^a^	0.133^a,b^	0.116^b^	0.130^a,b^	0.009	0.136^a^	0.085^b^	0.152^a^	0.155^a^	0.141^a^	0.010	0.045	<0.001	<0.001
***cis-9* C14:1**	0.293	0.269	0.330	0.278	0.033	0.263^c^	0.256^c^	0.301^b^	0.338^a^	0.303^b^	0.018	0.577	<0.001	0.953
***cis-9* C15:1**	0.104	0.100	0.094	0.111	0.006	0.113^a^	0.096^c^	0.103^b^	0.099^b,c^	0.099^b,c^	0.004	0.253	<0.001	0.261
***trans-4* C16:1**	0.029	0.034	0.032	0.033	0.002	0.027^b,c^	0.023^c^	0.031^b^	0.030^b^	0.050^a^	0.002	0.543	<0.001	0.597
***trans-5* C16:1**	0.030^f^	0.038^e,f^	0.039^e,f^	0.041^e^	0.003	0.025^c^	0.028^c^	0.024^c^	0.042^b^	0.066^a^	0.003	0.099	<0.001	0.150
***trans-6-7* C16:1**	0.045^c^	0.100^a^	0.059^b,c^	0.095^a,b^	0.013	0.061^b^	0.057^b^	0.070^b^	0.061^b^	0.126^a^	0.008	0.010	0.001	<0.001
***trans-9* C16:1**	0.076	0.083	0.067	0.077	0.007	0.044^c^	0.067^b^	0.096^a^	0.071^b^	0.100^a^	0.007	0.470	<0.001	<0.001
***trans-10* C16:1**	0.018^b^	0.026^b^	0.025^b^	0.045^a^	0.005	0.028^b^	0.024^b^	0.022^b^	0.023^b^	0.045^a^	0.003	0.007	<0.001	0.024
***trans-11-12* C16:1**	0.026^c^	0.044^ab^	0.039^b^	0.060^a^	0.005	0.055^a^	0.054^a^	0.041^b^	0.024^c^	0.038^b^	0.003	<0.001	<0.001	0.006
***cis-7* C16:1c7**	0.215^a^	0.152^b^	0.157^b^	0.138^b^	0.011	0.167^a,b^	0.171^a,b^	0.174^a^	0.157^b,c^	0.159^c^	0.007	<0.001	0.034	0.108
***cis-9* C16:1**	0.653^a^	0.458^b^	0.541^b^	0.415^b^	0.040	0.788^a^	0.773^a^	0.449^b^	0.255^d^	0.318^c^	0.025	0.001	<0.001	<0.001
***cis-10* C16:1**	0.029	0.032	0.033	0.043	0.005	0.047^a^	0.025^c^	0.031^b^	0.030^b^	0.037^b^	0.004	0.275	<0.001	0.208
***cis-11* C16:1**	0.033	0.034	0.035	0.046	0.005	0.029^c^	0.029^c^	0.037^b^	0.036^b^	0.054^a^	0.004	0.216	<0.001	0.143
***cis-6-7* C17:1**	0.023	0.023	0.025	0.023	0.002	0.029^a^	0.019^c^	0.020^c^	0.024^bc^	0.026^a,b^	0.002	0.595	<0.001	<0.001
***cis-8* C17:1**	0.029	0.025	0.026	0.028	0.002	0.021^b,c^	0.017^c^	0.020^b,c^	0.025^b^	0.052^a^	0.001	0.368	<0.001	<0.001
***cis-9* C17:1**	0.187^a^	0.128^b^	0.143^b^	0.127^b^	0.011	0.146	0.140	0.146	0.148	0.151	0.006	0.001	0.185	0.378
***trans-4* C18:1**	0.026^b^	0.038^a^	0.036^a^	0.036^a^	0.003	0.031	0.034	0.035	0.033	0.038	0.003	0.021	0.597	0.266
***trans-5* C18:1**	0.049^f^	0.061^e^	0.047^f^	0.049^f^	0.004	0.033^c^	0.033^c^	0.032^c^	0.085^a^	0.075^b^	0.004	0.057	<0.001	<0.001
***trans-6-8* C18:1**	0.185^b^	0.316^a^	0.225^b^	0.332^a^	0.026	0.278^a^	0.265^a,b^	0.277^a^	0.243^b^	0.257^a,b^	0.015	0.001	0.024	0.025
***trans-9* C18:1**	0.179^b^	0.364^a^	0.234^b^	0.386^a^	0.039	0.304^b^	0.294^b^	0.253^c^	0.262^b,c^	0.341^a^	0.023	0.002	<0.001	0.255
***trans-10* C18:1**	0.241^b^	0.614^a^	0.350^b^	0.662^a^	0.085	0.510^b^	0.415^b,c^	0.378^c^	0.418^b,c^	0.613^a^	0.049	0.004	0.002	0.001
***trans-11* C18:1**	0.485^b^	1.498^a^	0.579^b^	1.457^a^	0.264	0.946^b^	0.900^b^	0.723^b^	0.819^b^	1.635^a^	0.126	0.012	0.009	<0.001
***trans-12* C18:1**	0.209^b^	0.436^a^	0.294^b^	0.458^a^	0.040	0.359^b^	0.329^b,c^	0.317^c^	0.324^b,c^	0.417^a^	0.022	<0.001	<0.001	0.136
***trans-13-14* C18:1**	0.359^b^	0.558^a^	0.389^b^	0.524^a^	0.042	0.548^a^	0.451^b^	0.368^c^	0.366^c^	0.555^a^	0.028	0.004	<0.001	0.027
***cis-9* C18:1**	14.81^a^	10.62^b^	12.10^b^	10.62^b^	0.647	11.37^b^	12.39^a^	12.32^a^	11.87^a,b^	12.24^a^	0.359	<0.001	0.003	0.002
***cis-11* C18:1**	0.352^a^	0.307^a,b^	0.252^b^	0.311^a,b^	0.020	0.289^b^	0.286^b^	0.288^b^	0.289^b^	0.374^a^	0.012	0.013	0.001	0.079
***cis-12* C18:1**	0.196	0.168	0.159	0.175	0.012	0.165^b^	0.162^b^	0.180^a,b^	0.175^a,b^	0.190^a^	0.009	0.188	0.119	0.001
***cis-13* C18:1**	0.042^b^	0.065^a^	0.051^a,b^	0.065^a^	0.005	0.065^a^	0.052^b^	0.044^b^	0.044^b^	0.073^a^	0.004	0.007	<0.001	0.183
***cis-16-14* C18:1**	0.209^b^	0.318^a^	0.275^a^	0.330^a^	0.016	0.289^b^	0.267^b,c^	0.251^c^	0.282^b^	0.327^a^	0.012	<0.001	<0.001	0.050
***cis-16* C18:1**	0.046^b^	0.058^a^	0.053^a,b^	0.057^a^	0.003	0.026^c^	0.048^b^	0.065^a^	0.061^a^	0.067^a^	0.003	0.041	<0.001	<0.001
***cis-9* C20:1**	0.043	0.040	0.038	0.044	0.002	0.024^c^	0.026^c^	0.031^c^	0.044^b^	0.081^a^	0.002	0.315	<0.001	<0.001
***cis-11* C20:1**	0.025^b^	0.028^b^	0.035^a^	0.024^b^	0.002	0.032^e^	0.024^f^	0.025^f^	0.033^e^	0.025^f^	0.002	0.001	0.093	<0.001
***cis-15* C20:1**	0.032	0.039	0.033	0.035	0.002	0.035^a,b^	0.035^a,b^	0.031^b^	0.032^b^	0.040^a^	0.002	0.175	0.071	0.010
***cis-9* C22:1**	0.058^c^	0.112^a^	0.084^b^	0.059^c^	0.008	0.081^b^	0.172^a^	0.037^c^	0.024^c^	0.077^b^	0.008	<0.001	<0.001	0.005
***cis-15* C24:1**	0.028^f^	0.033^e,f^	0.035^e^	0.029^e,f^	0.002	0.024^b^	0.024^b^	0.028^b^	0.023^b^	0.059^a^	0.002	0.093	<0.001	0.245

1 CON: concentrate mix without microalgae; SC30: 30 g microalgae *Schizochytrium spp*./ewe/d; MB30: 30 microalgae blend g/ewe/d (21 g *Schizochytrium spp*. and 9 g *Nannochloropsis gaditana*); MB40: 40 microalgae blend g/ewe/d (28 g *Schizochytrium spp*. and 12 g *Nannochloropsis gaditana*).

2 SEM = Standard error of the mean.

Values are least squares means derived from a repeated-measures model. Measurements were obtained at five consecutive sampling times (d 18, 36, 54, 72, and 90 of the experimental period). Within each row, different lowercase superscript letters within the dietary treatment columns indicate differences among dietary treatments, whereas different lowercase superscript letters within the sampling time columns indicate differences among sampling days (*P* < 0.05). Dietary treatment means and sampling time means were compared separately. Lowercase superscript letters e and f indicate tendencies (0.05 ≤ *P* < 0.10) within the corresponding main effect.

Microalgae inclusion substantially modified PUFA profile (Table 7). The linoleic acid (C18:2 n-6) was unaffected (*P *= 0.444), whereas minor trans/cis isomers were altered by microalgae supplementation. Cis-9, trans-13 C18:2 and trans-8, cis-13 C18:2 increased in all supplemented groups compared with CON and cis-9, trans-12 C18:2 and trans-9, cis-12 C18:2 were increased in SC30 and MB40 compared to CON. Conjugated linoleic acid (CLA) isomers were strongly affected. The predominant cis-9, trans-11 CLA was significantly increased by SC30 and MB40 compared to CON. Conversely, trans-10, cis-12 CLA decreased in MB40 compared with CON (*P *= 0.043). Long-chain PUFA of marine origin showed the clearest treatment effects. EPA (C20:5 n-3) increased markedly (*P *< 0.001) with all microalgae supplementation, reaching the highest value with the MB40. DHA (C22:6 n-3) also rose significantly in all supplemented groups reaching the highest values in SC30 and MB40. In parallel, DPA (C22:5 n-6) increased nearly tenfold (*P *< 0.001) in all supplemented groups relative to CON.

**Table 7:** Polyunsaturated fatty acid composition (g/100 g FA) of milk from lactating ewes fed diets supplemented with microalgae.

	Dietary group^1^ (D)					Sampling time (T)						Effect		
	CON	SC30	MB30	MB40	SEM^2^	18	36	54	72	90	SEM^2^	D	T	D × T
***trans-10, trans-14* C18:2**	0.080^a^	0.064^b,c^	0.058^c^	0.067^b^	0.003	0.055^c^	0.080^a^	0.074^a,b^	0.070^b^	0.058^c^	0.003	<0.001	<0.001	<0.001
***trans-11, trans-15* C18:2**	0.021	0.024	0.027	0.020	0.003	0.024^b^	0.017^b^	0.018^b^	0.017^b^	0.037^a^	0.002	0.251	0.003	0.616
***trans-9, trans-12* C18:2**	0.027	0.028	0.032	0.031	0.003	0.030^b^	0.023^b,c^	0.027^b^	0.014^c^	0.053^a^	0.003	0.550	<0.001	0.103
***cis-9, trans-13* C18:2**	0.195^c^	0.307^a,b^	0.271^b^	0.336^a^	0.021	0.278^b^	0.248^c^	0.266^b,c^	0.283^b^	0.312^a^	0.013	<0.001	<0.001	0.091
***trans-8, cis-13* C18:2**	0.099^b^	0.130^a^	0.121^a^	0.137^a^	0.006	0.125^b^	0.108^c^	0.118^b^	0.124^b^	0.133^a^	0.004	0.001	<0.001	0.003
***cis-9, trans-12* C18:2**	0.061^c^	0.088^a^	0.071^b,c^	0.086^a,b^	0.006	0.090^a^	0.058^c^	0.070^b^	0.074^b^	0.090^a^	0.004	0.008	<0.001	0.004
***trans-9, cis-12* C18:2**	0.038^c^	0.063^a^	0.047^b,c^	0.059^a,b^	0.005	0.046^c^	0.045^c^	0.054^a,b^	0.052^b^	0.060^a^	0.003	0.005	0.022	0.001
***trans-11, cis-15* C18:2**	0.043^f^	0.059^e^	0.040^f^	0.049^e,f^	0.006	0.041^b^	0.042^b^	0.042^b^	0.042^b^	0.070^a^	0.004	0.092	0.023	0.001
**C18:2 n-6**	2.29	2.09	2.03	2.14	0.115	2.02^b^	2.02^b^	2.22^a^	2.19^a^	2.24^a^	0.065	0.444	<0.001	0.415
***trans-12, cis-15* C18:2**	0.073^e^	0.064^f^	0.071^e,f^	0.066^e,f^	0.003	0.050^c^	0.060^b^	0.076^a^	0.079^a^	0.076^a^	0.002	0.099	<0.001	0.001
**Δ7,9 17:2**	0.052^b^	0.075^a^	0.070^a^	0.058^b^	0.004	0.018^d^	0.042^c^	0.150^a^	0.087^b^	0.022^d^	0.007	0.002	<0.001	<0.001
**C18:3 n-6**	0.050^c^	0.076^b^	0.109^a^	0.096^a,b^	0.008	0.060^b^	0.051^b,c^	0.033^d^	0.038^c,d^	0.231^a^	0.008	<0.001	<0.001	<0.001
**C18:3 n-3**	0.411	0.403	0.403	0.436	0.026	0.396^b^	0.414^b^	0.441^a^	0.409^b^	0.406^b^	0.014	0.785	<0.001	0.417
***cis-9, trans-11* CLA**	0.351^b^	0.918^a^	0.456^b^	0.973^a^	0.156	0.624^b^	0.570^b^	0.538^b^	0.590^b^	1.050^a^	0.082	0.015	0.023	<0.001
***trans-9, cis-11* CLA/C21:0**	0.059^a^	0.061^a^	0.061^a^	0.049^b^	0.003	0.074^a^	0.070^b^	0.065^b^	0.051^c^	0.027^d^	0.002	0.006	<0.001	<0.001
***trans-10, cis-12* CLA**	0.023^a^	0.023^a^	0.019^a,b^	0.016^b^	0.002	0.024^a^	0.014^b^	0.018^b^	0.018^b^	0.026^a^	0.001	0.043	<0.001	0.002
***trans-12, trans-14* CLA**	0.030	0.030	0.034	0.026	0.003	0.024^c,d^	0.019^d^	0.045^a^	0.029^b,c^	0.034^b^	0.002	0.216	<0.001	0.149
***trans-11, trans-13* CLA**	0.027^b^	0.029^b^	0.037^a^	0.039^a^	0.003	0.048^a^	0.047^a^	0.021^c^	0.023^b,c^	0.027^b^	0.003	0.007	<0.001	<0.001
**C20:2 n-9**	0.022	0.025	0.027	0.024	0.002	0.028^b^	0.019^c^	0.021^c^	0.021^c^	0.034^a^	0.002	0.337	<0.001	0.337
***trans-9, trans-11* CLA**	0.041^b^	0.041^b^	0.041^b^	0.048^a^	0.002	0.026^c^	0.019^c^	0.040^b^	0.042^b^	0.088^a^	0.002	0.020	<0.001	<0.001
**C18:4 n-3**	0.022^e,f^	0.025^e,f^	0.027^e^	0.020^f^	0.002	0.022^b^	0.018^b^	0.027^a^	0.024^a,b^	0.027^a^	0.002	0.081	0.001	0.281
**C20:2 n-6**	0.039^a^	0.031^b^	0.031^b^	0.028^b^	0.002	0.032^b^	0.029^b^	0.040^a^	0.030^b^	0.030^a^	0.003	0.011	0.142	0.002
**C20:3 n-9**	0.038^a^	0.030^b^	0.029^b^	0.027^b^	0.003	0.039^a^	0.036^a,b^	0.024^c^	0.023^c^	0.031^b^	0.003	0.036	<0.001	0.002
**C20:3 n-6**	0.069^b^	0.087^a^	0.073^a,b^	0.084^a^	0.005	0.044^d^	0.075^c^	0.147^a^	0.095^b^	0.031^d^	0.005	0.049	<0.001	<0.001
**10,14,17 C20:3**	0.027^b^	0.039^a^	0.031^b^	0.032^b^	0.002	0.036^b^	0.029^b^	0.028^b^	0.020^c^	0.049^a^	0.003	0.008	<0.001	0.090
**C20:3 n-3**	0.230	0.260	0.236	0.242	0.010	0.175^c^	0.212^b^	0.265^a^	0.273^a^	0.285^a^	0.011	0.153	<0.001	<0.001
**C20:4 n-6**	0.134^b^	0.169^a^	0.144^b^	0.156^a,b^	0.007	0.085^c^	0.060^d^	0.192^b^	0.200^a,b^	0.215^a^	0.006	0.010	<0.001	<0.001
**C20:4 n-3**	0.018	0.027	0.021	0.013	0.004	0.011^c^	0.006^c^	0.014^b^	0.040^a^	0.028^b^	0.004	0.171	<0.001	0.002
**C22:2 n-6**	0.027	0.023	0.030	0.025	0.005	0.014^b,c^	0.011^c^	0.014^c^	0.030^b^	0.063^a^	0.005	0.663	<0.001	0.114
**C20:5 n-3**	0.061^c^	0.105^b^	0.093^b^	0.139^a^	0.007	0.090^b^	0.097^b^	0.106^a^	0.096^a,b^	0.107^a^	0.005	<0.001	0.038	<0.001
**C22:3 n-6**	0.015^e^	0.019^e^	0.016^e^	0.003^f^	0.004	0.004^c^	0.007^b,c^	0.011^b,c^	0.017^a,b^	0.027^a^	0.004	0.099	0.058	0.114
**C22:4 n-6**	0.709	0.717	0.751	0.671	0.031	0.367^d^	0.549^c^	0.788^b^	0.893^a^	0.964^a^	0.030	0.355	<0.001	<0.001
**C22:5 n-6**	0.031^c^	0.346^a^	0.240^b^	0.341^a^	0.022	0.225^b^	0.250^a^	0.259^a^	0.236^a,b^	0.228^b^	0.013	<0.001	0.005	<0.001
**C22:5 n-3**	0.087^b^	0.119^a^	0.095^b^	0.125^a^	0.008	0.103^b^	0.099^b^	0.106^b^	0.103^b^	0.121^a^	0.005	0.009	0.001	0.354
**C22:6 n-3**	0.046^c^	1.090^a^	0.779^b^	1.130^a^	0.076	0.653^c^	0.785^a,b^	0.839^a^	0.776^b^	0.767^b^	0.041	<0.001	<0.001	0.026

1 CON: concentrate mix without microalgae; SC30: 30 g microalgae *Schizochytrium spp*./ewe/d; MB30: 30 microalgae blend g/ewe/d (21 g *Schizochytrium spp*. and 9 g *Nannochloropsis gaditana*); MB40: 40 microalgae blend g/ewe/d (28 g *Schizochytrium spp*. and 12 g *Nannochloropsis gaditana*).

2 SEM = Standard error of the mean.

Values are least squares means derived from a repeated-measures model. Measurements were obtained at five consecutive sampling times (d 18, 36, 54, 72, and 90 of the experimental period). Within each row, different lowercase superscript letters within the dietary treatment columns indicate differences among dietary treatments, whereas different lowercase superscript letters within the sampling time columns indicate differences among sampling days (*P* < 0.05). Dietary treatment means and sampling time means were compared separately. Lowercase superscript letters e and f indicate tendencies (0.05 ≤ *P* < 0.10) within the corresponding main effect. CLA: conjugated linoleic acids.

Grouped FA classes are reported in Table 8. SCFA increased (*P *= 0.002) in SC30 and MB40 compared with CON. Similarly, medium-chain fatty acids (MCFA) were higher in MB30 relative to CON (*P *= 0.047), whereas long-chain fatty acids (LCFA) decreased significantly (*P *= 0.007) in all microalgae-fed ewes. Total PUFA content was higher in SC30 and MB40 than in CON (*P *< 0.001), whereas MB30 exhibited an intermediate value. The n-3 PUFA content more than doubled (*P *< 0.001) in SC30 and MB40 compared with CON whereas MB30 exhibited an intermediate value. Further, n-6 PUFA were not affected by microalgae supplementation (*P *= 0.598), and consequently, the n-3/n-6 ratio increased significantly in all treated groups compared to CON.

**Table 8:** Grouped fatty acid profile (g/100 g FA) of milk from lactating ewes fed diets supplemented with microalgae.

	Dietary group (D)^1^					Sampling time (T)						Effect		
	CON	SC30	MB30	MB40	SEM^2^	18	36	54	72	90	SEM^2^	D	T	D × T
**SCFA**	17.76^b^	19.92^a^	18.52^b^	20.49^a^	0.488	20.30^a^	19.81^a,b^	19.42^b^	19.00^c^	17.34^d^	0.285	0.002	<0.001	0.800
**MCFA**	46.66^b^	46.94^b^	49.06^a^	46.03^b^	0.753	47.47^b^	46.93^b^	47.12^b^	48.55^a^	45.81^c^	0.420	0.047	<0.001	0.121
**LCFA**	8.42^a^	6.16^b^	6.69^b^	6.23^b^	0.473	7.07^a,b^	7.29^a^	6.78^b^	6.00^c^	7.22^a,b^	0.303	0.007	0.001	0.001
**BCFA**	1.79	1.67	1.85	1.83	0.074	1.59^c^	1.50^d^	1.76^b^	2.04^a^	2.06^a^	0.045	0.318	<0.001	<0.001
**MUFA**	19.60^a^	17.38^b^	17.02^b^	17.39^b^	0.505	17.46^c^	18.12^b^	17.47^c^	16.96^d^	19.23^a^	0.278	0.005	<0.001	0.466
**PUFA**	5.56^c^	7.69^a^	6.63^b^	7.80^a^	0.350	5.99^c^	6.24^c^	7.20^b^	7.12^b^	8.05^a^	0.194	<0.001	<0.001	0.001
**n-3**	1.04^c^	2.22^a^	1.82^b^	2.28^a^	0.101	1.60^c^	1.78^b^	1.96^a^	1.88^a^	1.97^a^	0.060	<0.001	<0.001	0.083
**n-6**	3.52	3.76	3.60	3.73	0.145	3.05^d^	3.20^c^	3.87^b^	3.89^b^	4.26^a^	0.077	0.598	<0.001	0.006
**n-3/n-6**	0.30^c^	0.59^a^	0.51^b^	0.61^a^	0.017	0.52^b^	0.55^a^	0.51^b^	0.48^c^	0.47^c^	0.010	<0.001	<0.001	0.006
**SFA**	72.84	73.03	74.27	72.76	0.764	74.85^a^	74.04^b^	73.31^c^	73.56^b,c^	70.38^d^	0.416	0.476	<0.001	0.082
**UFA**	25.16	25.07	23.64	25.18	0.748	23.44^c^	24.36^b^	24.67^b^	24.08^b,c^	27.28^a^	0.410	0.403	<0.001	0.115
**SFA/UFA**	2.94	2.97	3.17	2.92	0.115	3.22^a^	3.07^b,c^	3.00^c^	3.10^b^	2.61^d^	0.064	0.408	<0.001	0.254

1 CON: concentrate mix without microalgae; SC30: 30 g microalgae *Schizochytrium spp*./ewe/d; MB30: 30 microalgae blend g/ewe/d (21 g *Schizochytrium spp*. and 9 g *Nannochloropsis gaditana*); MB40: 40 microalgae blend g/ewe/d (28 g *Schizochytrium spp*. and 12 g *Nannochloropsis gaditana*).

2 SEM = Standard error of the mean.

Values are least squares means derived from a repeated-measures model. Measurements were obtained at five consecutive sampling times (days 18, 36, 54, 72, and 90 of the experimental period). Within each row, different lowercase superscript letters within the dietary treatment columns indicate differences among dietary treatments, whereas different lowercase superscript letters within the sampling time columns indicate differences among sampling days (*P* < 0.05). Dietary treatment means and sampling time means were compared separately. SCFA, short-chain fatty acids; MCFA, medium-chain fatty acids; LCFA, long-chain fatty acids; SFA, refers to straight-chain SFA, BCFA are reported separately; UFA, unsaturated fatty acids; MUFA, monounsaturated fatty acids; PUFA, polyunsaturated fatty acids; BCFA, branched-chain fatty acids. SFA = SCFA + MCFA + LCFA; PUFA = total n-6 + total n-3 + CLA, and other PUFA isomers. Short-chain saturated fatty acids (SCFA) = C4:0 + C6:0 + C7:0 + C8:0 + C9:0 + C10:0 + C11:0; medium-chain saturated fatty acids (MCFA) = C12:0 + C13:0 + C14:0 + C15:0 + C16:0; long-chain saturated fatty acids (LCFA) = C17:0 + C18:0 + C19:0 + C20:0 + C22:0 + C23:0 + C24:0 + C25:0 + C26:0.

The transfer efficiencies of DHA and C22:5 n-6 averaged 41.6% and 40.5%, respectively, across the microalgae-supplemented treatments, whereas EPA transfer efficiency averaged 39.2% in the blend treatments, where EPA intake was increased. No significant differences in fatty acid transfer efficiency were observed among dietary groups. Furthermore, milk from ewes fed the SC30, MB30, and MB40 diets was enriched with 72.5, 51.4, and 77.5 mg/100 g of EPA + DHA, respectively (data not shown).

### Blood and milk oxidative status

Dietary supplementation with microalgae affected several biomarkers of the antioxidant system and oxidative status in both blood plasma and milk of lactating ewes (Table 9). In plasma, no significant differences among dietary treatments were observed for FRAP, ABTS, SOD, GSH-Px, GR, or MDA (*P *> 0.10). However, CAT activity tended to increase (*P *= 0.066) with higher levels of microalgae supplementation, reaching the greatest value in ewes fed the MB40 diet (114.0 U/mL) compared with CON (60.08 U/mL). GST activity was affected by dietary treatment (*P *= 0.036). MB40 showed the greatest value (0.064 U/mL) and was higher than SC30 (0.047 U/mL), whereas CON (0.049 U/mL) and MB30 (0.059 U/mL) showed intermediate values. Protein carbonyl (PC) concentration was significantly affected by dietary treatment (*P *= 0.015), showing a reduction in MB30 (4.03 nmol/mL) compared with CON (4.41 nmol/mL) and SC30 (4.49 nmol/mL).

**Table 9:** Blood plasma and milk total antioxidant capacity, antioxidant enzyme activities, and oxidative stress biomarkers of lactating ewes fed diets supplemented with microalgae.

	Dietary group (D)^1^					Sampling time (T)						Effect		
	CON	SC30	MB30	MB40	SEM^2^	18	36	54	72	90	SEM^2^	D	T	D × T
**Blood plasma**														
**FRAP**	0.67	0.69	0.71	0.76	0.045	0.74^a^	0.67^b^	0.75^a^	0.67^b^	0.70^a^	0.029	0.550	<0.001	0.308
**ABTS**	38.61	39.02	37.90	37.75	0.701	36.98^b^	38.20^a,b^	38.53^a,b^	39.70^a^	38.18^a,b^	0.481	0.539	<0.001	0.486
**SOD**	8.12	8.28	7.83	9.09	0.548	7.26	8.31	8.65	8.48	8.95	0.459	0.502	0.074	0.021
**GSH-Px**	0.37	0.38	0.39	0.36	0.022	0.37^b^	0.33^c^	0.40^a^	0.40^a^	0.36^b^	0.013	0.772	<0.001	0.004
**CAT**	60.08^f^	74.68^e,f^	76.16^e,f^	114.00^e^	13.405	71.44	81.91	89.62	78.03	85.16	8.284	0.066	0.100	0.051
**GST**	0.049^a,b^	0.047^b^	0.059^a,b^	0.064^a^	0.004	0.046^c^	0.054^a,b^	0.058^a,b^	0.064^a^	0.050^b,c^	0.004	0.036	0.010	0.138
**GR**	0.023	0.024	0.025	0.025	0.002	0.023^c^	0.021^d^	0.021^c,d^	0.026^b^	0.031^a^	0.001	0.723	<0.001	0.138
**PC**	4.41^a^	4.49^a^	4.03^b^	4.19^a,b^	0.103	4.11^b,c^	4.12^bc^	4.86^a^	4.05^c^	4.26^b^	0.088	0.015	<0.001	<0.001
**MDA**	0.79	0.84	0.80	0.86	0.038	0.85^a^	0.83^a,b^	0.79^b^	0.86^a^	0.79^b^	0.024	0.599	0.020	0.401
**Milk**														
**FRAP**	3.66	2.19	3.20	2.00	0.598	3.12^a^	2.96^a,b^	2.69^b,c^	2.70^b^	2.34^c^	0.276	0.172	0.001	0.669
**ABTS**	33.61^a^	29.93^b^	27.95^b^	27.64^b^	1.116	28.87^a,b^	25.80^b^	30.43^a,b^	35.77^a^	28.04^a,b^	0.693	0.003	<0.001	<0.001
**SOD**	102.63	94.28	100.07	92.54	8.54	92.87	100.62	97.84	102.37	93.19	6.94	0.783	0.370	0.104
**GSH-Px**	0.595^a^	0.386^b^	0.357^b^	0.381^b^	0.060	0.420	0.385	0.434	0.493	0.416	0.045	0.043	0.295	0.770
**CAT**	5.17	4.76	4.79	4.52	0.690	4.93	4.90	5.11	4.48	4.62	0.505	0.920	0.853	0.532
**PC**	2.90^e,f^	3.14^e,f^	2.83^f^	3.22^e^	0.112	2.76^b^	3.06^a^	3.35^a^	3.36^a^	2.57^b^	0.133	0.062	0.001	0.058
**MDA**	0.54^b,c^	0.59^a^	0.52^b^	0.57^a,b^	0.015	0.60^a^	0.54^b^	0.57^b^	0.56^b^	0.48^c^	0.012	0.016	<0.001	<0.001

1 CON: concentrate mix without microalgae; SC30: 30 g microalgae *Schizochytrium spp*./ewe/d; MB30: 30 microalgae blend g/ewe/d (21 g *Schizochytrium spp*. and 9 g *Nannochloropsis gaditana*); MB40: 40 microalgae blend g/ewe/d (28 g *Schizochytrium spp*. and 12 g *Nannochloropsis gaditana*).

2 SEM = Standard error of the mean.

Values are least squares means derived from a repeated-measures model. Measurements were obtained at five consecutive sampling times (d 18, 36, 54, 72, and 90 of the experimental period). Within each row, different lowercase superscript letters within the dietary treatment columns indicate differences among dietary treatments, whereas different lowercase superscript letters within the sampling time columns indicate differences among sampling days (*P* < 0.05). Dietary treatment means and sampling time means were compared separately. Lowercase superscript letters e and f indicate tendencies (0.05 ≤ *P* < 0.10) within the corresponding main effect. FRAP, ferric reducing antioxidant power (μM ascorbic acid); ABTS radical scavenging activity (% inhibition); SOD, superoxide dismutase (U/mL); GSH-Px, glutathione peroxidase (U/mL); CAT, catalase (U/mL); GST, glutathione-S-transferase (U/mL); GR, glutathione reductase (U/mL); PC, protein carbonyls (nmol/mL); MDA, malondialdehyde (μM).

In milk, total antioxidant capacity (ABTS, *P *= 0.003) and GSH-Px activity (*P *= 0.043) were significantly influenced by dietary treatment. The ABTS radical scavenging capacity was highest in CON milk (33.61% inhibition) and significantly reduced in all microalgae-fed groups (27.6%-29.9%). Conversely, GSH-Px activity was highest in the CON group (0.595 U/mL) and significantly decreased in all microalgae-fed ewes (0.357-0.386 U/mL), with no difference among the supplemented groups. Milk PC concentration tended to be affected (*P *= 0.062), with MB40 showing a higher value (3.22 nmol/mL) compared with MB30 (2.83 nmol/ml). Milk MDA concentration was affected by dietary treatment (*P *= 0.016), with the highest value observed in SC30 (0.59 μM) and the lowest in MB30 (0.52 μM), whereas MB40 (0.57 μM) showed an intermediate value and CON (0.54 μM) did not differ from MB30 or MB40.

## Discussion

### Nutrient intake

Feed and nutrient intake were mostly unaffected by microalgae inclusion. Daily feed refusals consisted only of alfalfa hay. This contrasts with earlier findings in goats, where higher *Schizochytrium spp*. supplementation (40-60 g/goat/d) reduced feed consumption (Mavrommatis and Tsiplakou 2020). The absence of a similar response here may be explained by the isoenergetic and isolipidic formulation, because microalgae replaced part of soybean and palm oil rather than being added on top of the diet, together with the lower supplementation levels used here. Regarding FA intake, microalgae supplementation increased long-chain PUFA intake compared with CON, reflecting the known FA profiles of *Schizochytrium spp*. and *N. gaditana* as DHA- and EPA-rich sources, respectively (Ma et al. 2016; Sahin et al. 2018).

### Milk yield and chemical composition

The inclusion of *Schizochytrium* and *Nannochloropsis* microalgae in ewe diets only slightly altered milk fat content while having no effect on milk yield, as reported in other ruminant studies (Franklin et al. 1999; Mavrommatis and Tsiplakou 2020; Zisis et al. 2022). A tendency toward lower milk fat percentage was observed in MB30, suggesting only a weak indication of MFD. This finding is consistent with reports that high dietary inclusion of long-chain n-3 PUFA (especially from marine sources) can induce MFD in sheep and goats (Toral et al. 2010; Bichi et al. 2013; Mavrommatis and Tsiplakou 2020). Interestingly, the reduction in milk fat content was observed in the group receiving the lower level of PUFA. This differential response may reflect a non-linear treatment response in ruminal biohydrogenation dynamics and/or mammary lipid metabolism. However, it should be emphasized that this reduction represented only a statistical trend, with no significant difference between MB30 and MB40. Therefore, these changes cannot be classified as a clear MFD, at least not to the extent previously reported in sheep and goats, where milk fat decreased by 21% and 33%, respectively (Mavrommatis and Tsiplakou 2020; Zisis et al. 2022). However, it should be acknowledged that the moderate number of animals per treatment may have limited the statistical power to detect some effects.

### Milk fatty acid profile

Dietary supplementation with microalgae, particularly *Schizochytrium spp*., increased the proportion of SCFA (C6:0, C8:0, and C10:0) in ewe milk. This finding contrasts with previous studies in sheep and goats, in which SCFA concentrations were not affected by dietary supplementation with marine-derived PUFA (Boeckaert et al. 2008; Bichi et al. 2013; Mavrommatis and Tsiplakou 2020). With regard to BCFA, no changes were observed in total BCFA concentration; however, the proportions of iso-C17:0 and iso-C13:0 increased in response to the microalgae blends containing *N. gaditana*. These results may suggest that the inclusion levels and the combination of microalgae used in this study did not adversely affect ruminal fermentation, despite the potential inhibitory effects of PUFA on ruminal microorganisms previously reported by Maia et al. (2010) and Mavrommatis et al. (2021a). The preservation of milk BCFA may indicate a well-functioning rumen environment, typically dominated by cellulolytic bacterial species (Fievez et al. 2012).

In milk, C14:0 tended to be lower in CON than in the microalgae-fed groups, whereas C16:0 was higher in MB30 than in SC30 and MB40, with CON showing an intermediate value. Because milk C14:0 is predominantly synthesized *de novo* in the mammary gland, the tendency toward greater C14:0 proportions in the supplemented groups cannot be explained by its greater dietary supply. This response may instead reflect treatment-related changes in mammary *de novo* fatty acid synthesis. Similarly, although milk C16:0 originates from both dietary uptake and mammary *de novo* synthesis, its higher proportion in MB30 despite lower dietary intake may indicate altered mammary fatty acid synthesis (Chilliard et al. 2000; Kyriakaki et al. 2022). However, because mammary lipogenic activity was not directly assessed, these interpretations remain speculative. From a nutritional perspective, the increase in C16:0 and the tendency toward higher C14:0 in MB30 should be interpreted cautiously, as both are LDL-cholesterol-raising SFA, and higher intakes have been associated with greater coronary heart disease risk (Mensink et al. 2003; Zong et al. 2016; Sellem et al. 2022). However, current guidance emphasizes replacing SFA with UFA within the overall dietary pattern, while the cardiometabolic effects of dairy fat may also depend on the dairy food matrix (Sacks et al. 2017; WHO 2023; Taormina et al. 2024).

Reduced milk C18:0 has been reported in ewes (Papadopoulos et al. 2002; Zisis et al. 2022), cows (Franklin et al. 1999), and goats (Fougère et al. 2018; Mavrommatis and Tsiplakou 2020) fed *Schizochytrium spp*.-enriched diets. In agreement with these findings, milk C18:0 concentration was reduced in the present study; however, the magnitude of this decrease was milder than in the aforementioned studies. In previous studies, pronounced inhibition of ruminal biohydrogenation has typically been associated with MFD, whereas no severe MFD was observed in the present experiment. A similar response was reported by Pajor et al. (2021), who observed reduced milk C18:0 in goats supplemented with *Schizochytrium spp.* without concurrent MFD, likely due to the relatively low inclusion level used. This also contrasts with our previous study in dairy ewes supplemented with the same dose of *Schizochytrium spp*. (30 g/ewe/d), in which a much stronger inhibition of biohydrogenation was observed (Zisis et al. 2022). This difference may be partly related to the lactation stage. Previous studies have reported that trans-C18:1 isomers increase as lactation progresses, reflecting a greater extent of incomplete ruminal biohydrogenation (Kay et al. 2005). In parallel, the abundance of *Butyrivibrio spp*., a ruminal biohydrogenating bacterium, has been shown to decrease as lactation advances in dairy cows (Bainbridge et al. 2016). Together, these findings suggest that biohydrogenation efficiency may be higher in early than in later lactation. Because the present study commenced in early lactation and extended into mid-lactation, ruminal biohydrogenation may have been more complete than in our previous trial, contributing to the higher milk C18:0 concentration and the absence of severe MFD despite microalgae supplementation. It should also be acknowledged that monitoring changes in biohydrogenation-related milk fatty acid intermediates before the first sampling point would have been informative. However, milk qualitative characteristics were not assessed at day 0 in the present study, limiting our ability to describe the earliest changes following the onset of dietary supplementation.

In this context, the results concerning C18:0 indicate an inhibition of the final step of the biohydrogenation process in the rumen of ewes, as also evidenced by the significant increase in trans-C18:1 isomers in milk. In particular, trans-10 C18:1 concentrations increased significantly in ewes fed the SC30 and MB40 diets. Interestingly, the trans-10 C18:1 isomer, previously associated with antilipogenic effects in the mammary gland (Shingfield et al. 2009), did not differ significantly in the MB30 group, where a tendency toward lower milk fat content was observed. This finding further complicates the interpretation, as trans-10, cis-12 CLA was also unaltered in the MB30 group. Similar increases in trans-C18:1 isomers have been reported in dairy ewes supplemented with marine lipid sources, including marine algae or fish oil, and have generally been interpreted as evidence of incomplete ruminal biohydrogenation of dietary unsaturated fatty acids (Papadopoulos et al. 2002; Toral et al. 2010; Bichi et al. 2013; Carreño et al. 2016). In particular, previous studies with marine algae in dairy ewes showed that trans-10 C18:1 accumulation may accompany MFD, whereas increases in trans-11 C18:1 are often associated with greater cis-9, trans-11 CLA formation (Bichi et al. 2013). Therefore, the lack of a significant increase in trans-10 C18:1 and trans-10, cis-12 CLA in MB30 suggests that the tendency for lower milk fat percentage in this group cannot be explained solely by these specific biohydrogenation intermediates but may reflect a broader and treatment-dependent pattern of ruminal lipid metabolism.

Although most trans-C18:1 isomers increased in the milk of microalgae-fed ewes, the overall MUFA concentration decreased significantly across all treatments due to the lower proportion of oleic acid (cis-9 C18:1). The reduction in C18:0 may partly account for the decline in total MUFA and particularly cis-9 C18:1, because a substantial proportion of milk cis-9 C18:1 is formed endogenously in the mammary gland through the activity of stearoyl-CoA desaturase (Δ^9^-desaturase), which converts C18:0 to cis-9 C18:1 (Chilliard et al. 2000). Moreover, the activity of Δ^9^-desaturase has been shown to decrease when PUFA are incorporated into ruminant diets (Clarke and Jump 1994), with marine-derived PUFA (particularly EPA and DHA) exerting a stronger inhibitory effect on mammary stearoyl-CoA desaturase (SCD) expression and activity than plant-derived PUFA (Kyriakaki et al. 2022). At this point, it is important to note that although other studies reported a greater reduction in milk C18:0, as previously discussed, our results are more comparable when considering the decline in cis-9 C18:1. Specifically, inclusion of *Schizochytrium* spp. in ruminant diets has been shown across multiple studies to decrease milk C18:0 by an average of approximately 67%, with reported reductions ranging from 56 to 80%. In comparison, the present study showed a more moderate reduction of 22.7%–30.2%. In contrast, reductions in cis-9 C18:1 reported across different studies average around 40% (range: 20%–62%), which is more closely aligned with the decrease of up to 28.3% observed in the current study (Franklin et al. 1999; Boeckaert et al. 2008; Toral et al. 2010; Bichi et al. 2013; Mavrommatis and Tsiplakou 2020; Zisis et al. 2022).

In the present study, the transfer efficiencies of DPA, EPA, and DHA to milk were higher than those reported in previous studies supplementing PUFA in ruminant diets. More specifically, as summarized by Farina et al. (2015), transfer efficiency to goat milk ranges from 7.0% to 14.0% for EPA and from 7.0% to 8.1% for DHA when rumen-unprotected fish oil is included in the diet. Farina et al. (2015) also summarized that, when algal supplements were used, DHA transfer efficiency in dairy cows was 8.4% with unprotected algae and 16.7% with rumen-protected forms. Similarly, in sheep, DHA transfer efficiency from unprotected algal sources ranged between 5.0% and 8.0%, whereas 30%–60% of dietary EPA was incorporated into milk. In the present study, the combined dietary supply of n-6 DPA and DHA ranged from 45 to 64 mg/kg BW. This was lower than the dietary supply reported in the sheep study summarized by Farina et al. (2015), in which a DHA transfer efficiency of 5% was associated with a supplementation level of approximately 140 mg n-6 DPA plus DHA/kg BW. These findings collectively indicate a clear negative correlation between dietary intake of long-chain PUFA and their transfer efficiency into milk. The response observed in MB40 supports the complementary rationale of the blend, as this treatment provided both a high *Schizochytrium spp*. supply and *N. gaditana* and resulted in the greatest increase in milk EPA. Therefore, identifying an optimal inclusion level may not only mitigate adverse effects on MFD but also improve the economic efficiency of microalgae supplementation through enhanced transfer efficiency.

### Oxidative balance in milk and ewes

Dietary supplementation with microalgae rich in PUFA (DHA, EPA, and n-6 DPA) at high inclusion levels has been shown to induce oxidative stress through the activation of NADPH oxidase. More specifically, in our previous study, dietary supplementation of goats with 40 and 60 g *Schizochytrium spp*./goat/d increased the enzymatic activity of NADPH oxidase in blood plasma (Mavrommatis et al. 2018). Additionally, in monocytes, the relative transcript levels of *NOX1* and *NOX2* tended to be upregulated with 40 and 60 g *Schizochytrium spp*./goat/d (Kyriakaki et al. 2023). In neutrophils, both *NOX1* and *NOX2* were significantly upregulated only at 60 g *Schizochytrium spp*./goat/d, demonstrating the importance of PUFA inclusion level in oxidative manifestation (Kyriakaki et al. 2023). Considering all data from our previous studies with *Schizochytrium spp*. in goats and ewes, we concluded that supplementation providing more than 70 mg of DHA and n-6 DPA from *Schizochytrium spp*./kg BW in small ruminants can induce excessive ROS production through NOX activation impairing antioxidant mechanisms (Mavrommatis et al. 2018; Zisis et al. 2022; Kyriakaki et al. 2023). In the present study, supplementation remained within this upper bound and was not associated with marked increases in the main oxidative damage biomarkers, particularly MDA and PC. Based on this evidence, the PUFA levels used across dietary treatments did not appear to markedly activate the main ROS-generating systems. This is further supported by findings from our recent study within the same experimental trial, where microalgae supplementation, particularly in MB30-fed ewes, was associated with lower *NOX2* mRNA levels in blood neutrophils (Mavrommatis et al. 2026).

In blood plasma, we observed an enhancement of the enzymatic antioxidant defenses. Both CAT and GST activities showed higher values with greater microalgae inclusion; CAT tended to be greatest in MB40, whereas GST was greatest in MB40 and differed from SC30, with CON and MB30 showing intermediate values. SOD constitutes the first line of defense against oxidative stress, protecting cells from damage by detoxifying the superoxide anion, while CAT and GSH-Px form the second line of defense by catalyzing the conversion of hydrogen peroxide to water and oxygen and the reduction of peroxides to alcohols and water (Palma et al. 2020; Jomova et al. 2023). GST is responsible for the metabolic inactivation of electrophilic compounds, including lipid hydroperoxides (Board and Menon 2013; Labrou et al. 2015). Overall, the combination of microalgae (MB30) showed lower protein oxidation in plasma (reduced PC levels) than control. Improved enzymatic defenses, together with lower PC, partially support our hypothesis that combining microalgae, including an antioxidant-rich strain (El-Sayed et al. 2024), can alleviate the oxidative response associated with the high susceptibility of PUFA to oxidation.

In milk, the antioxidant response was more variable. The total antioxidant capacity of milk (ABTS assay) and milk GSH-Px activity were lower in microalgae-fed ewes than in CON. This likely reflects the fact that milk with more unsaturated fat is more prone to oxidation; unsaturated lipids and proteins can consume antioxidants, lowering measurable capacity (Smet et al. 2009). Notably, MB30 showed the lowest milk MDA value, whereas SC30 showed the highest value and MB40 was intermediate. Milk PC tended to be lower in MB30 than in MB40, while CON and SC30 showed intermediate values. Therefore, MB30 showed the most favorable response for some oxidative biomarkers, particularly milk MDA and PC, although the overall antioxidant response in milk remained variable. One possible explanation is that the exogenous antioxidant compounds from the microalgae blend were sufficient at 30 g/d to protect milk lipids, but at 40 g/d the increase in PUFA began to generate more peroxides than enzymes could neutralize. This aligns with Zisis et al. (2022), who found that 30 g/d *Schizochytrium* was well tolerated, whereas 40 g/d increased milk MDA and PCs in ewes.

Overall, in milk, the ABTS radical-scavenging capacity and GSH-Px activity decreased, hinting at local consumption of antioxidants by PUFA oxidation. Importantly, none of the treatments produced a consistent increase in both MDA and PC, suggesting that microalgae supplementation did not induce marked oxidative damage under the conditions of the present study. Thus, we hypothesized that the natural antioxidant complements in *Schizochytrium* and *Nannochloropsis* could be sufficient to counteract the peroxidation of oxidation-prone PUFAs, provided that supplementation remains moderate. These findings corroborate previous work: in goats, adding 40–60 g/d of *Schizochytrium* caused pronounced oxidative stress (higher MDA, lower antioxidant enzymes), but 20 g/d had negligible negative effect (Mavrommatis et al. 2018). The present study suggests that similar effects occur in sheep and that blending *Nannochloropsis* may partially mitigate oxidative risk due to its rich antioxidant profile (El-Sayed et al. 2024).

## Conclusions

In conclusion, dietary supplementation with *Schizochytrium spp*. alone or combined with *N. gaditana* enriched ewe milk with long-chain n-3 PUFA without impairing milk yield or major production traits. Importantly, the combined microalgae treatments enriched milk with the dominant fatty acids supplied by each microalga, namely DHA from *Schizochytrium spp*. and EPA from *N. gaditana*, indicating that the tested blend can improve the nutritional fatty acid profile of milk in a complementary manner. Nevertheless, this response should be interpreted together with the observed changes in SFA, particularly C14:0 and C16:0, and the variable oxidative-status responses. Overall, the results support the use of microalgae supplementation as a dietary strategy to produce n-3-enriched ewe milk, while highlighting the need to optimize the dose and blend composition to maximize beneficial fatty acid enrichment and minimize unfavorable shifts in milk fat composition.

## Data Availability

The data underlying this article are available in the article and in its online supplementary material.

## Abbreviations

ABTS: 2,2′-azino-bis(3-ethylbenzthiazoline-6-sulfonic acid)
BCFA: branched-chain fatty acids
CAT: catalase
CLA: conjugated linoleic acid
DHA: docosahexaenoic acid
DPA: docosapentaenoic acid
ECM: energy-corrected milk
EPA: eicosapentaenoic acid
FCM: fat-corrected milk
FA: fatty acid
FAME: fatty acid methyl ester
FRAP: ferric reducing antioxidant power
GSH-Px: glutathione peroxidase
GR: glutathione reductase
GST: glutathione transferase
LCFA: long-chain fatty acids
MCFA: medium-chain fatty acids
MDA: malondialdehyde
MFD: milk fat depression
MUFA: monounsaturated fatty acids
PC: protein carbonyls
PUFA: polyunsaturated fatty acids
ROS: reactive oxygen species
SCFA: short-chain fatty acids
SFA: saturated fatty acids
SOD: superoxide dismutase
